# “Adjusting internal organs and dredging channelon” electroacupuncture glycolipid metabolism disorders in NAFLD mice by mediating the AMPK/ACC signaling pathway

**DOI:** 10.1186/s13098-024-01416-7

**Published:** 2024-07-25

**Authors:** Xinyu Jia, Mengyuan Li, Wen Zhang, Yihui Guo, Fuyu Xue, Shiqi Ma, Shuo Yu, Zhen Zhong, Haipeng Huang

**Affiliations:** 1 College of Traditional Chinese Medicine, Changchun University of Chinese Medicine, Changchun, 130117 Jilin China; 2 Department of Northeast Asian Center for Traditional Chinese Medicine, Changchun University of Chinese Medicine, Changchun, 130117 Jilin China; 3 The Fourth Clinical Medical College of Guangzhou University of Chinese Medicine, Shenzhen, 518033 China; 4 Department of Endocrinology, Shenzhen Traditional Chinese Medicine Hospital, Shenzhen, 518033 China; 5 Acupuncture and Massage Treatment Center, The Third Affiliated Clinical Hospital of Changchun University of Chinese Medicine, Changchun, 130117 Jilin China; 6 Department of Institute of Acupuncture and Massage, College of Acupuncture and Massage, Changchun University of Chinese Medicine, No.1035 Boshuo Road, Changchun, 130117 Jilin China

**Keywords:** Type 2 diabetes mellitus with nonalcoholic fatty liver disease, AMPK/ACC signaling pathway, Glucolipid metabolism disorder, Electrocupuncture, Insulin resistance

## Abstract

To investigate the effect mechanism of electroacupuncture based on the AMP-activated protein kinase (AMPK) /acetyl-CoA carboxylase (ACC) signaling pathway to improve glycolipid metabolism disorders in db/db mice. 10 db/m mice with normal genotype were used as the normal control group without diabetes (Con), and 30 db/db mice were divided randomly into three groups: Pathological model mice (Mod), Acupuncture + ACC antagonist group (Acu + ACC), and Acupuncture + AMPK antagonist group (Acu + AMPK). Con and Mod did not receive any special treatment, only as a control observation. The latter two groups of mice received electroacupuncture treatment for 4 weeks. Mouse triglyceride (TG), high-density lipoprotein cholesterol (HDL-C), low-density lipoprotein cholesterol(LDL-C), and cholesterin(CHO) levels were detected by colorimetric assay. Enzyme-linked immunoassay (ELISA) was used to detect insulin(INS) levels. Liver histopathologic changes and hepatic glycogen synthesis were observed by HE and PAS staining. The mRNA and protein expression of insulin receptor substrate-1(IRS1), Phosphatidylinositol 3-kinase(PI3K), protein kinase B (AKT), AMPK, and ACC were detected by Western blot and qRT-PCR.The results show that compared with Mod, TG, LDL, CHO, and INS levels of Acu + AMPK and Acu + ACC mice were significantly reduced (*P* < 0.05), and the HDL levels were significantly increased (*P* < 0.05), the steatotic degeneration of mice hepatocytes was reduced to different degrees, and the hepatocyte glycogen particles were increased, and the latter two groups had a decrease in AKT, ACC mRNA expression was reduced (*P* < 0.05), PI3K protein expression was increased, and AKT and ACC protein expression was reduced (*P* < 0.05), in addition, protein expression of AMPK was increased and IRS1 protein expression was reduced in Acu + ACC (*P* < 0.05). The study showed that electroacupuncture improves glucose-lipid metabolism disorders in db/db mice, and this mechanism is related to the AMPK/ACC signaling pathway.

## Introduction

Glycolipid metabolism disorder is characterized by alterations in glucose and lipid metabolism, and the main pathological manifestations are neuroendocrine disorders, insulin resistance (IR), oxidative stress, altered inflammatory response, imbalance of intestinal flora, and abnormalities of glucose and lipids are often accompanied by metabolic disorders of the internal environment, which are accompanied by the emergence of diseases such as non-alcoholic fatty liver disease (NAFLD), hypertension, atherosclerosis, and so on [1]. The incidence of metabolic diseases has increased sharply, which has brought major treatment challenges and a huge economic burden to global public health [2]. According to the latest data from the International Diabetes Federation (IDF), there will be 537 million people with diabetes worldwide, accounting for 10.5% of the world’s population [3]; more than 90% of patients are diagnosed with type 2 diabetes mellitus (T2DM) [4, 5], and about 70–75% of these patients experience NAFLD [6–8]. Patients with NAFLD in combination with T2DM not only have a high risk of developing diabetes mellitus but also have a high risk of developing complications. T2DM patients with NAFLD are not only a high-risk group for diabetes complications, but also a priority group for the prevention and treatment of hepatocellular carcinoma and cirrhosis [9]. Therefore, it is particularly urgent to seek a treatment method with high patient satisfaction, accurate efficacy, and long-lasting effects by introducing the idea of Chinese medicine.

It has been found that the mechanisms of T2DM with NAFLD include IR [10], hepatic free fatty acid (FFA) accumulation [11], oxidative stress imbalance [12], abnormal expression of insulin-like growth factor-1 (IGF-1) [13], and endoplasmic reticulum stress (ERS) [14], etc., but these mechanisms have yet to be further elucidated. Currently, modern medical treatment of T2DM combined with NAFLD is mainly based on strict dietary control and pharmacologic therapy with thiazolidinediones [15], metformin [16], glucagon-like receptor agonists GLP-1RA [17] and α-glucosidase inhibitors [18]. Most modern treatments are used to treat T2DM combined with NAFLD using lowering blood glucose levels; there are no targeted medications for this disease, and the side effects and cost of treatment associated with long-term medication can be extremely burdensome to patients’ health and finances. In comparison to modern medical treatment, several studies have demonstrated that acupuncture treatment in traditional Chinese medicine effectively improves blood glucose and dyslipidemia in patients with abnormalities of glucose and lipid metabolism [19, 20]. Acupuncture can regulate hepatic fat accumulation in patients and animal models [21, 22] and regulate hepatic gene expression [23]. Acupuncture has a positive regulatory effect on glycolipid metabolism, and its mechanism involves multiple signaling pathways and multiple targets. Acupuncture is widely used in the adjuvant treatment of a variety of metabolic diseases, but the detailed biological explanation of acupuncture stimulation is still limited [2]. The liver is the main organ of glycolipid metabolism, and its mechanism is closely related to the pathway abnormalities of glycolipid metabolism. Therefore, there is an urgent need to elucidate the mechanism of acupuncture intervention in T2DM with combined NAFLD.

In our previous study, we found that liver IR exists in db/db mice, insulin signaling depends on Insulin receptor substrate-1 (IRS1), phosphorylated IRS1 will lead to the activation of insulin signaling, and when IR occurs in the liver, it also reduces the insulin sensitivity, so that the insulin signaling pathway is impaired, the expression of IRS tyrosine phosphorylation and the disappearance of Phosphatidylinositol 3-kinase(PI3K) and protein kinase B(AKT), electroacupuncture can be used to regulate liver PI3K-Akt in db/db mice through the regulation of PI3K-Akt. The impairment of the insulin signaling pathway reduces insulin activity, the expression of IRS tyrosine phosphorylation, and the disappearance of PI3K and Akt. Electroacupuncture can affect glucose-lipid metabolism by regulating the expression of key proteins of the PI3K-Akt-GSK3β signaling pathway in the liver of db/db mice [24], which fully confirms that electroacupuncture can improve the level of IR in the liver of db/db mice. We found that AMPK/ACC is the key target of electroacupuncture to regulate this disease in the preliminary experiments, AMP-activated protein kinase (AMPK) is the receptor of cellular energy metabolism, and the activation of hepatic AMPK can regulate the balance of cellular glucose and energy metabolism, and acetyl-CoA carboxylase (ACC) is a rate-limiting enzyme to promote the synthesis of fatty acids. In addition to abnormal glucose-lipid metabolism, fatty acid synthesis is reduced, suggesting that ACC is regulated by insulin and affects fatty acid synthesis [25], and ACC has gradually become a potential target for the treatment of T2DM with NAFLD [26].ACC is a downstream target of AMPK, and in the pathological environment of high fat and high glucose, downregulation of AMPK activates the activity of the downstream target of ACC, exacerbating IR, and thereby In high-fat and high-glucose pathologies, AMPK downregulation will activate the activity of the downstream target ACC, exacerbate IR, and thus aggravate the condition of T2DM with NAFLD [27, 28]. Therefore, the impairment of signaling pathways targeting AMPK/ACC can aggravate glucose metabolism disorder on the one hand, and lead to the accumulation of fatty acids and lipid metabolism abnormality on the other hand.

The research began by examining the interaction between glycolipids and the liver’s IR-mediated AMPK/ACC signaling pathway. The focus was on uncovering the potential mechanism behind the effectiveness of electroacupuncture in treating T2DM with NAFLD. The study’s findings offer new ideas and a theoretical basis for treating glycolipid metabolism disorders in clinical Chinese medicine and acupuncture.

## Methods

### Animal and ethics statement

All animal experiments were conducted in the Animal Experiment Center of Changchun University of Traditional Chinese Medicine. 30 male db/db mice of SPF grade at 6 weeks of age, weighing 30 ± 2 g, and 10 male db/m mice of the same litter, weighing 20 ± 2 g, were used. Db/db mice carry mutations in the Leptin gene, which shows obesity, insulin resistance, hyperglycemia, fatty liver, and other symptoms in mice. Although db/m mice carry pathogenic genes, their phenotypes remain normal. Mice were obtained from Jiangsu Jicui Pharmachem (Jiangsu, China; License No. SCXK (Su) 2023-0009). The temperature in the animal cages was controlled at 23 ± 1 °C, relative humidity 50-70%, and all mice were kept in cages alternating light and dark for 12 h each, with the noise lower than 60 dB. The mice were maintained on mouse maintenance diets (60% carbohydrates, 22% proteins, and 4.0% fats) in separate cages of 5 mice per cage, with free access to water. The study was approved by the Ethics Committee of Changchun University of Traditional Chinese Medicine (approval number: 2,023,386). All experiments were conducted by the Guidelines for the Breeding and Use of Laboratory Animals at Changchun University of Traditional Chinese Medicine.

### Animal treatment

Before the start of the experiment, the mice were acclimatized and reared for one week. Subsequently, the animals were grouped for modeling. 30 db/db mice were randomly assigned to a model control group (Mod), an acupuncture + ACC antagonist group (Acu + ACC), and an acupuncture + AMPK antagonist group (Acu + AMPK), with 10 mice in each group. Additionally, 10 db/m mice kept under the same conditions were used as the normal control group (Con). Db/m mice in the Con group did not receive any experimental treatment but were only observed as normal controls to observe the physiological status of mice under normal conditions. The mod group is the pathological state of db/db mice after successful modeling, but without experimental treatment, it is only used as model observation to observe the natural course of the model. At the baseline, the researchers measured the body weight, water intake, food intake, and blood glucose levels in the mice. Outliers were removed based on these results.

During the experimental period, all four groups of mice were given the same food and water for a fixed period. Electroacupuncture treatment was administered to Acu + ACC mice and Acu + AMPK mice once a day for 20 min per session, for a total of six sessions per session, and consecutive interventions for four sessions. The specific acupoints selected for acupuncture treatment were BL13 (Lung Yu), BL20 (Spleen Yu), BL23 (Kidney Yu), LI4 (Hegu), LR3 (Taichong), ST36 (Sansanli), and SP6 (Sanyinjiao), with bilateral acupoints chosen. These acupoints were referenced from the acupuncture point atlas for mice in the national 13th Five-Year Plan textbook Experimental Acupuncture and Moxibustion (Beijing: China Press of Traditional Chinese Medicine, 2016). Acupuncture procedures were performed using 0.18 × 10 mm needles from Suzhou Medical Instrument Factory in Suzhou, China. The depth of the needle varied according to the acupuncture point: 4 mm for BL13, BL20, and BL23; 3 mm for ST36; 1.5 mm for SP6; and 1 mm for LI4 and LR3. Electroacupuncture treatments were performed using unilateral BL13-BL23 and SP6-ST36 as acupuncture points. The SDZ-V electroacupuncture instrument of the Huatuo brand was used for electroacupuncture stimulation with a continuous wave at a frequency of 3 Hz. The intensity was set at a level that the muscles in the stimulated area could contract slightly and the mice could tolerate it. The treatment time was 20 min. Mice in Con and Mod were used as control observations only.

During the 4-week treatment period, we measured the mice’s body weight, water and food intake, and blood glucose levels weekly. Before blood glucose measurement, the mice underwent a 12-hour fast without water. We collected blood samples by pricking the tail end of the mice with a disposable sterile needle. After the blood flowed out, we measured the blood glucose levels of each mouse using a Roche blood glucose meter and accompanying test paper.

### Sample collection

After the determination of the basic indexes, mice were anesthetized by inhalation of isoflurane, and blood was extracted from the medial canthus. The blood was allowed to stand at room temperature for 30 min, then centrifuged at 3000 rpm/min for 15 min at 4 °C. The upper layer of serum was extracted and stored in freezing tubes at -80 °C. The blood was then stored in a refrigerator at -80 °C for reserve. After blood collection, rat livers were quickly extracted, washed in ice saline, and weighed after the surface water was absorbed by filter paper. Two pieces of liver tissue were cut from the right lobe of the liver at the same position of the outer 1/3 and outer 2/3, placed in an embedding box, and fixed in a 4% neutral formaldehyde solution. The remaining liver specimens were put into freezing tubes, quick-frozen in liquid nitrogen, and then stored in a -80 °C refrigerator for spare parts for the analysis of physiological and pathological indexes. Finally, the mice were executed by cervical dislocation.

### Measurement of biochemical indicators

Serum levels of TG, LDL-C, HDL-C, and CHO indexes were measured using an automatic serum biochemical analyzer.

### Enzyme-linked immunoassay (ELISA) for INS indicators

The ELISA analysis was used to determine the serum insulin level (INS) of each group of mice using serum specimens.

The mouse FINS enzyme-linked immunosorbent assay kit (Hai Jianglai Biotechnology Co., Ltd., Product No. JL11459)was used to detect serum insulin (INS).

### HE and PAS

The liver tissue was embedded in paraffin, sectioned, and stained with Hematoxylin and Eosin (HE) staining. The liver lesions were then observed and photographed under a 10x light microscope.

PAS staining is generally used to show sugars such as glycogen in tissues, and PAS-positive cytoplasm is red. Methods: after routine paraffin embedding, sectioning, and staining with Schiff’s solution, the liver tissue was observed under a light microscope, and the red color of the cytoplasm was regarded as a positive reaction, and vice versa was regarded as a negative reaction.

### Western blot analysis

Proteins from the liver of mice were extracted using the 1% Nonidet-P40 (NP-40) method [29]. The resulting lysate contained Tris 50 mM, NaCl 150 mM, and ethylene diamine tetraacetic acid(EDTA) 15 mM (pH = 8.0). The lysate was mixed and stored at 4 °C for later use. After determining the protein concentration using the bicinchoninic acid assay(BCA) method, an equal amount of protein was loaded onto a 10% sodium dodecyl sulfate – polyacrylamide gel electrophoresis (SDS-PAGE gel), electrophoresed, and transferred to a polyvinylidene liuoride (PVDF) membrane. The transfer of the pre-stained protein marker on the PVDF membrane was used to judge the protein transfer. After being sealed with 5% skimmed milk powder for 1 h at room temperature, the PVDF membrane was gently rinsed with 1 x tris buffered saline (TBST). The primary antibody was then diluted with 11 antibody diluent containing 5% bovine albumin (BSA) and incubated overnight at 4 °C. The next day, the PVDF membrane was incubated with 1 x TBST, and the primary antibody was again diluted with 11 antibody diluents containing 5% BSA. The membrane was washed three times with 1 x TBST for 10 min each time on the following day. Then, the corresponding secondary antibody was diluted with 5% BSA antibody diluent according to 1. The membrane was incubated with 10,000 for 1 h at room temperature on a shaker. After incubation, the membrane was washed three times with 1 x TBST for 10 min each time. Finally, the luminescent substrate Electrochemiluminescence (ECL) reaction solution was used, and the proteins were visualized by scanning the film with a scanner after development and fixation. Finally, the luminescent substrate ECL reaction solution was used, and the proteins were visualized by scanning the film with a scanner after development and fixation.

### Detection of mRNA expression of IRS1, PI3K, AKT, AMPK and ACC by qRT-PCR

Mouse liver tissues were collected from each group and analyzed for mRNA expression levels of IRS1, PI3K, AKT, AMPK, and ACC using real-time fluorescence quantitative PCR. Hepatocyte total RNA was extracted using Trizol reagent (Thermo Fisher Scientific, USA) following the procedures outlined in the RT-PCR kit manual. The cDNA was obtained through reverse transcription of RNA using a reverse transcription kit (Thermo Fisher Scientific, USA) as a template. Real-time quantitative polymerase chain reaction (QPCR) was used to detect the cDNA, with the following amplification conditions: pre-denaturation at 95℃ for 10 min, followed by 40 cycles of 95℃ for 15 s and 60℃ for 60 s. It is important to repeat this step to confirm the accuracy of the reaction conditions and to pay attention to the trend direction of the amplification curve. Data Analysis: The mRNA expression levels of the gene of interest were calculated using the 2^−ΔΔCT^ relative quantification method. No changes in content were made. The gene’s absorbance values were tested using the GelDoc2000 gel image analyzer, and the corresponding internal reference GAPDH was used to determine the gene’s relative mRNA expression.

### Statistical analysis

The data were analyzed using Statistical Products and Services Solution (SPSS). Graphs were created using GraphPad Prism 8. Measurements were expressed as mean ± standard deviation (SD). The statistical significance of the differences between the four groups was calculated using one-way analysis of variance (ANOVA). *p* < 0.05 was considered statistically significant.

## Results

### Changes in body weight, food intake, and water intake of mice in each group

**Fig. 1:**
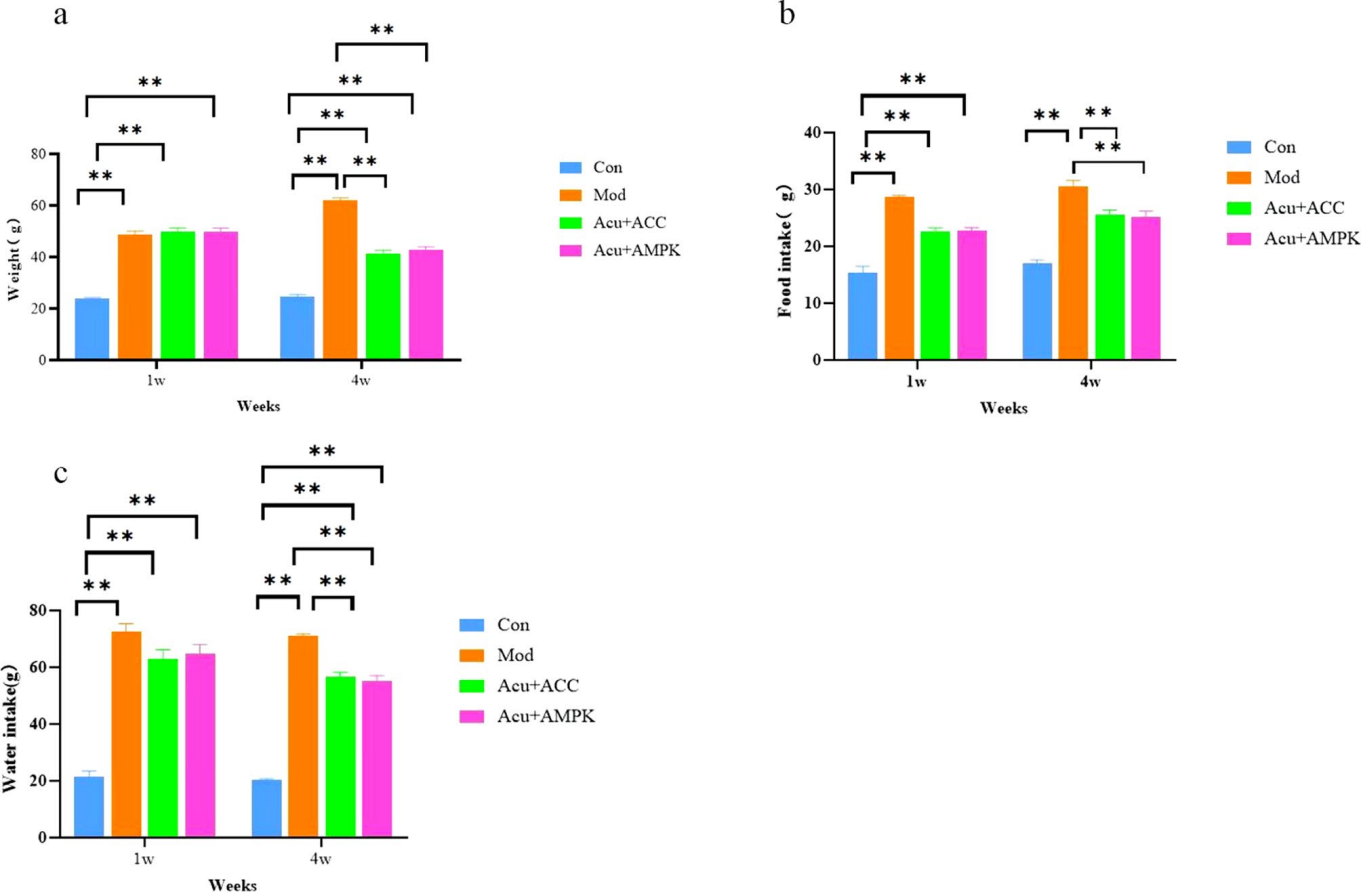
Comparison of general conditions of mice in each group. (**a**)Body weight. (**b**)Food intake. (**c**)Water intake. Results are mean value ± SD(*n* = 5).**p < 0.01,compared with Con.**p < 0.01,compared with Mod.**p < 0.01,compared with Acu + ACC

At 1 week of intervention, the body weight, food intake, and water intake of mice in the Mod group increased significantly compared with those in the Con group (*P* < 0.01), and there was no difference between the mice in the Mod group and those in the Acu + AMPK antagonist group and the Acu + ACC antagonist group (*P* > 0.05); at 4 weeks of intervention, the changes in body weight of mice in the Mod group were significantly different from those of mice in the Acu + AMPK antagonist group and the Acu + ACC antagonist group, the body weight, food intake and water intake of mice in the Mod group were significantly reduced, with significant differences (*P* < 0.01) (Fig. 1). The above results indicated that Acu + AMPK antagonist and Acu + ACC antagonist could improve the body weight gain, drinking and eating more in db/db mice to a certain extent, and had a better effect.

### Changes in blood glucose of mice in each group

**Fig. 2:**
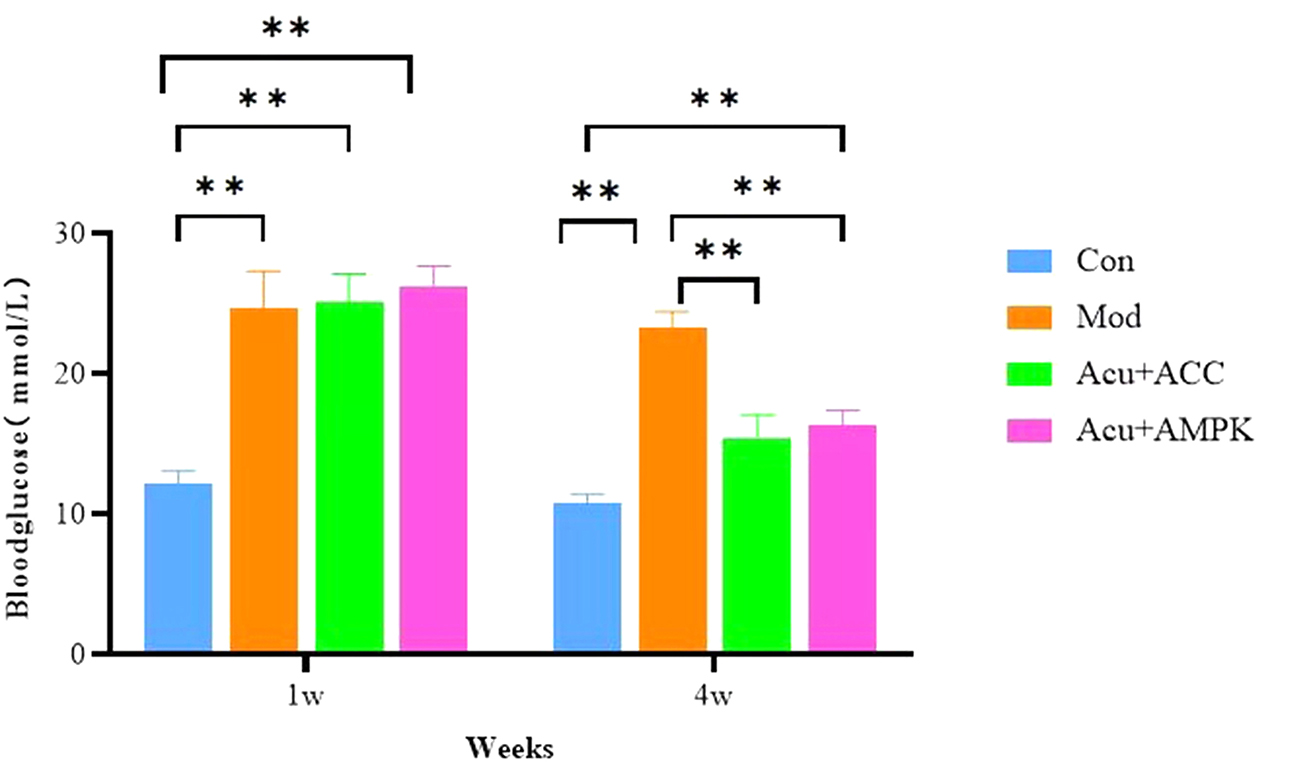
Changes in blood glucose in each group of mice. Results are mean value ± SD(*n* = 5).**p < 0.01,compared with Con.**p < 0.01,compared with Mod.**p < 0.01,compared with Acu + ACC

At 1 week of intervention, compared with the mice in the Con group, the blood glucose of the mice in the Mod group increased significantly (*P* < 0.05), and there was no significant difference between the mice in the Mod group and the Acu + AMPK antagonist group and the Acu + ACC antagonist group (*P* > 0.05); at 4 weeks of intervention, compared with the mice in the Con group, there was a significant blood glucose improvement (*P* < 0.05) (Fig. 2). The above results indicate that Acu + AMPK antagonist and Acu + ACC antagonist can improve the blood glucose elevation of db/db mice to some extent with significant efficacy.

### Comparison of TG, LDL, HDL, and CHO levels in mice in each group

**Fig. 3:**
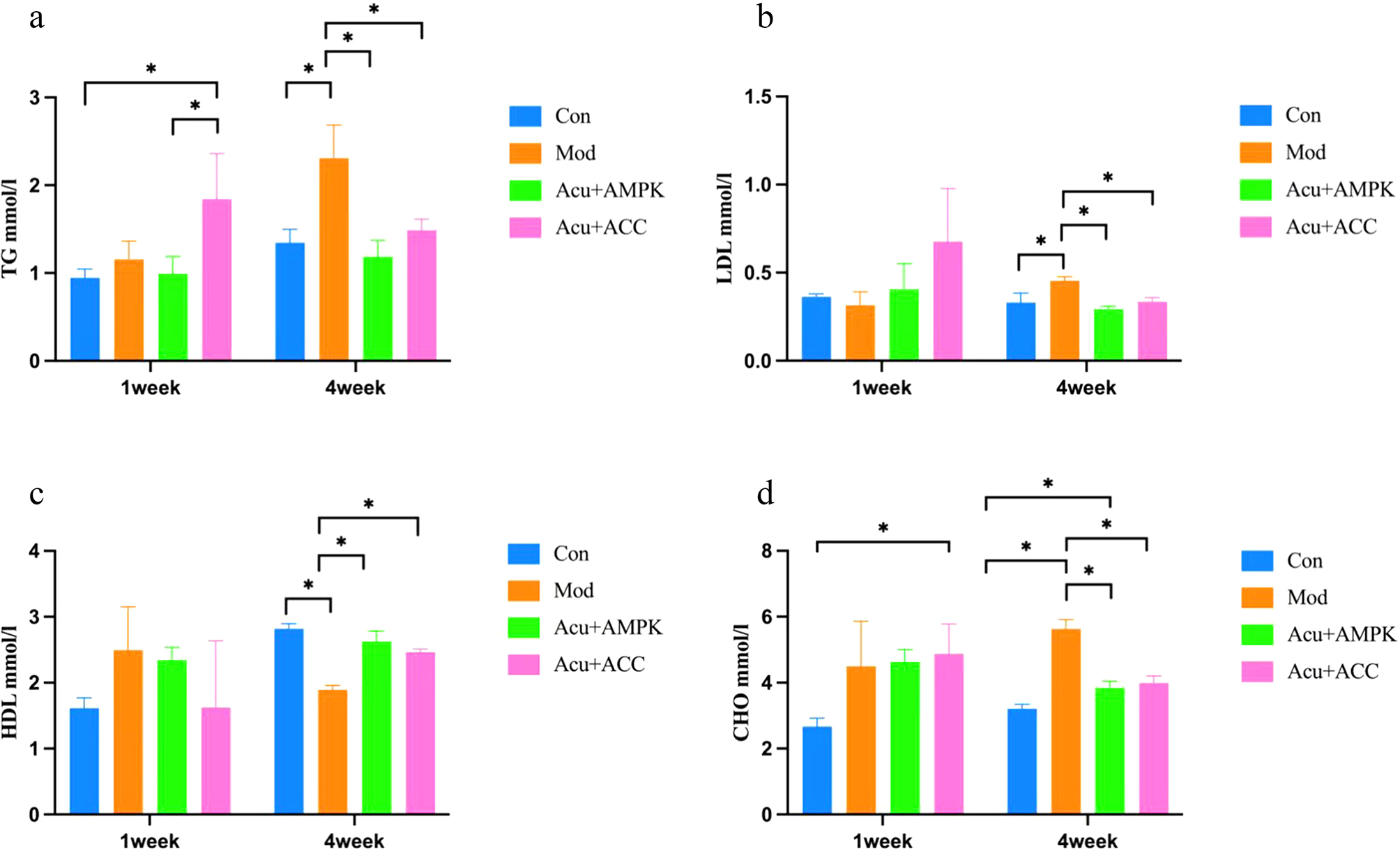
Comparison of biochemical indices of mice in each group. (**a**)TG: Triglycerides. (**b**)LDL-C: low-density lipoprotein. (**c**)HDL-C: high-density lipoprotein cholesterol. (**d**)CHO: total cholesterol. Results are mean value ± SD(*n* = 5).*p < 0.05,compared with Con.*p < 0.05,compared with Mod.*p < 0.05,compared with Acu + ACC

Compared with mice in the Con group, mice in the Mod group had significantly higher levels of TG, LDL, CHO (*P* < 0.05) and lower levels of HDL (*P* < 0.05); compared with mice in the Mod group, mice in the Acu + AMPK antagonist group had significantly lower levels of TG, LDL, CHO (*P* < 0.05) and higher levels of HDL (*P* < 0.05), the mice in Acu + ACC antagonist group showed significant improvement in all the above indexes (*P* < 0.05) (Fig. 3). The above results indicate that when IR occurs in the liver, glucose-lipid metabolism is abnormal, and the indexes of the Acu + AMPK antagonist group still improve, which indicates that acupuncture treatment itself is effective and that Acu + ACC antagonist has therapeutic significance for NAFLD.

### Comparison of INS levels of mice in each group

**Fig. 4:**
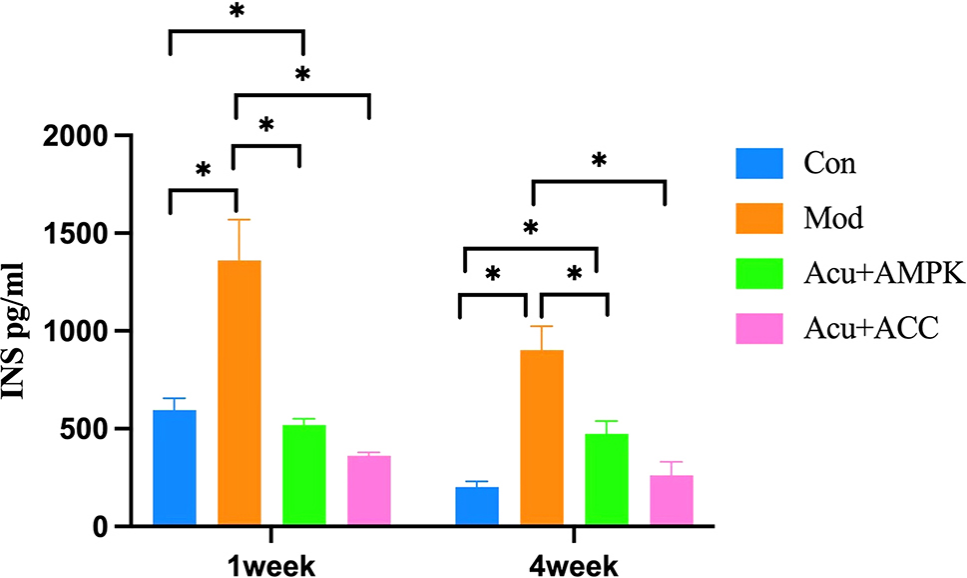
Comparison of serum insulin (INS) levels in mice in each group. Results are mean value ± SD(*n* = 5).*p < 0.05,compared with Con.*p < 0.05,compared with Mod.*p < 0.05,compared with Acu + ACC

Compared with the mice in the Con group, the INS levels of mice in the Mod group were significantly higher (*P* < 0.05); compared with the mice in the Mod group, the INS levels of mice in the Acu + AMPK antagonist group and Acu + ACC antagonist group were significantly decreased, with significant difference (*P* < 0.05) (Fig. 4). The above results indicated that the Acu + AMPK antagonist and Acu + ACC antagonist could significantly improve IR and attenuate serum INS elevation due to IR in db/db mice.

### Comparison of pathological conditions of mice in each group

HE staining results showed that: In Con, liver lobules were intact, hepatocytes were normal polygonal, nuclei were located in the center, uniform size, homogeneous staining of cytoplasm, and there was no significant steatosis; in Mod group, liver lobules were structurally abnormal, hepatocytes were enlarged and ballooned, and the whole liver tissue was diffusely steatotic; compared with the Mod group, the steatotic degeneration of hepatocytes in mice in the Acu + AMPK group and the Acu + ACC group was reduced to varying degrees.

**Fig. 5:**
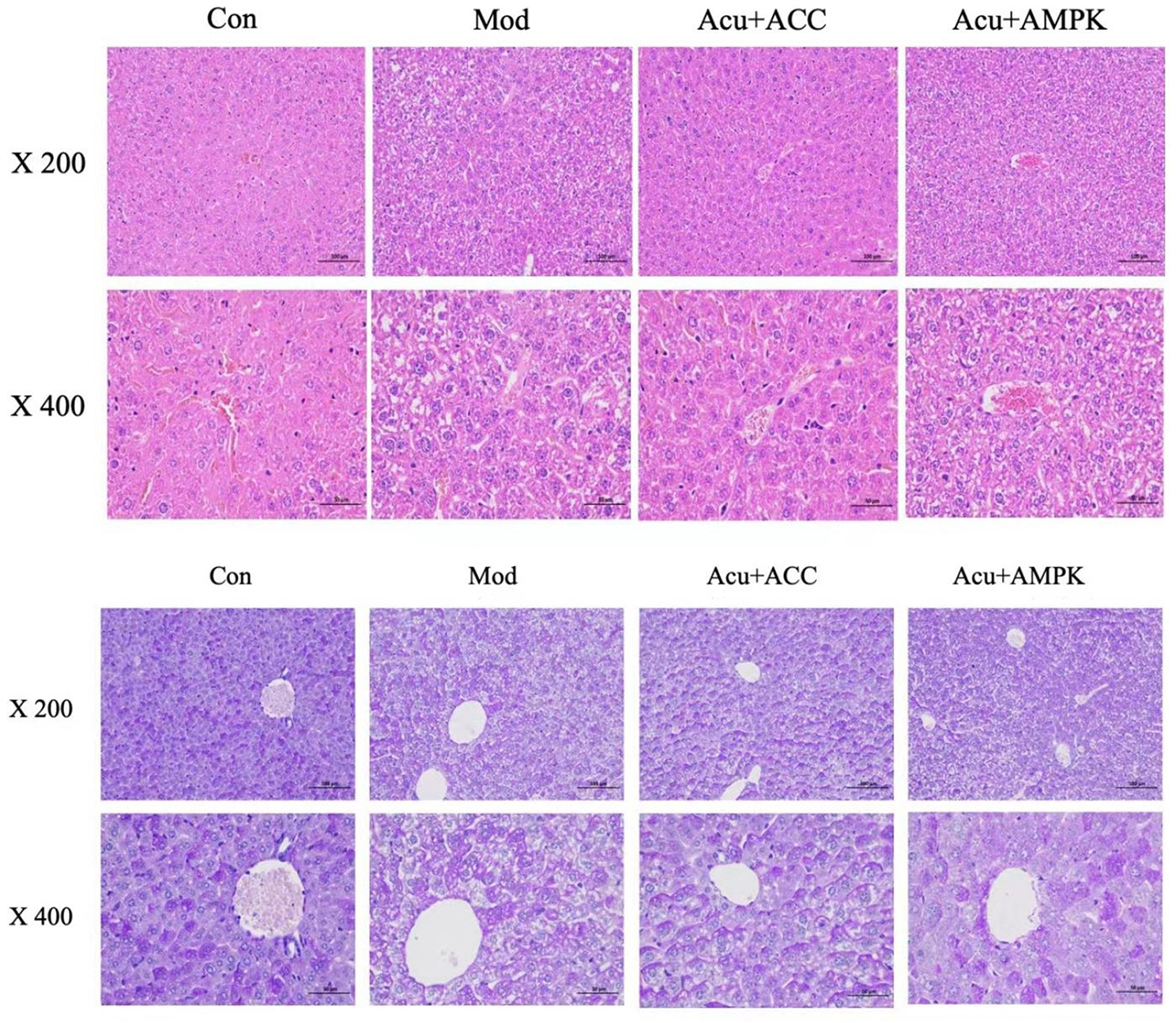
Comparison of pathological indices of mice in each group. (**a**)HE staining of liver tissue after intervention in each group of mice (200x/400x). (**b**)PAS staining of liver tissue after intervention in each group of mice (200x/400x)

PAS staining results showed that: Hepatocytes in the Con group were rich in intracytoplasmic glycogen particles, uniformly distributed, deeply stained, and with clear intercellular boundaries; hepatocytes in the Mod group showed obvious steatosis, cell swelling, and the distribution of intracytoplasmic glycogen particles was significantly reduced compared with that in the Con group. Compared with the Mod group, glycogen particles increased in the hepatocytes of Acu + AMPK and Acu + ACC groups, but the distribution of glycogen particles in the hepatocytes was not uniform (Fig. 5).

### Comparison of mRNA expression levels of IRS1, PI3K, and AKT.AMPK, and ACC in the liver tissues of mice in various groups

Detection results of mRNA expression level of IRS1. At 1 week of intervention, the mRNA expression of IRS1 in the Acu + ACC antagonist group was increased compared with the Mod group, with a significant difference (*P* < 0.05).

Detection results of mRNA expression level of PI3K. At 1 week of intervention, the mRNA expression of PI3K was increased in the Mod group compared with the Con group, with a significant difference (*P* < 0.05).

Detection results of mRNA expression level of AKT. At 4 weeks of intervention, compared with the Con group, the mRNA expression of AKT in the Mod group increased, with a highly significant difference (*P* < 0.01); compared with the Mod group, the mRNA expression of AKT in the Acu + AMPK antagonist group and the Acu + ACC antagonist group decreased, with significant difference (*P* < 0.05); compared with the Acu + AMPK antagonist group, the Acu + ACC antagonist group, the mRNA expression of AKT was increased with significant difference (*P* < 0.05).

Results of mRNA expression level of AMPK. At 4 weeks of intervention, the mRNA expression of AMPK was increased in the Acu + ACC antagonist group compared with the Con group, with a significant difference (*P* < 0.05).

**Fig. 6:**
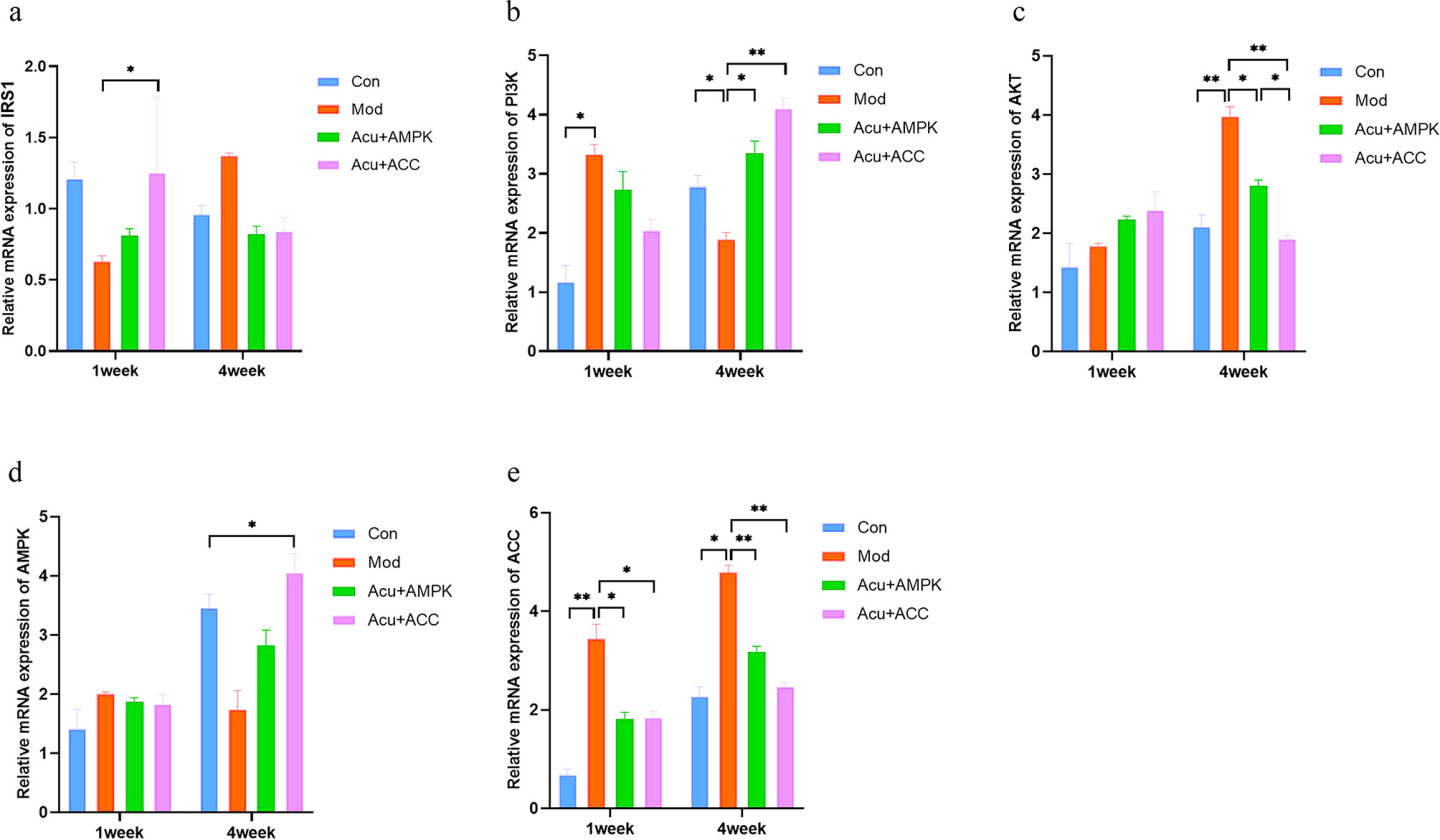
Comparison of mRNA expression levels of IRS1(a), PI3K(b), AKT(c), AMPK(d), and ACC(e) in liver tissues of mice in each group. Results are mean value ± SD(*n* = 5).*p < 0.05,**p < 0.01,compared with Con.*p < 0.05,**p < 0.01,compared with Mod.*p < 0.05,**p < 0.01,compared with Acu + ACC

**Fig. 7:**
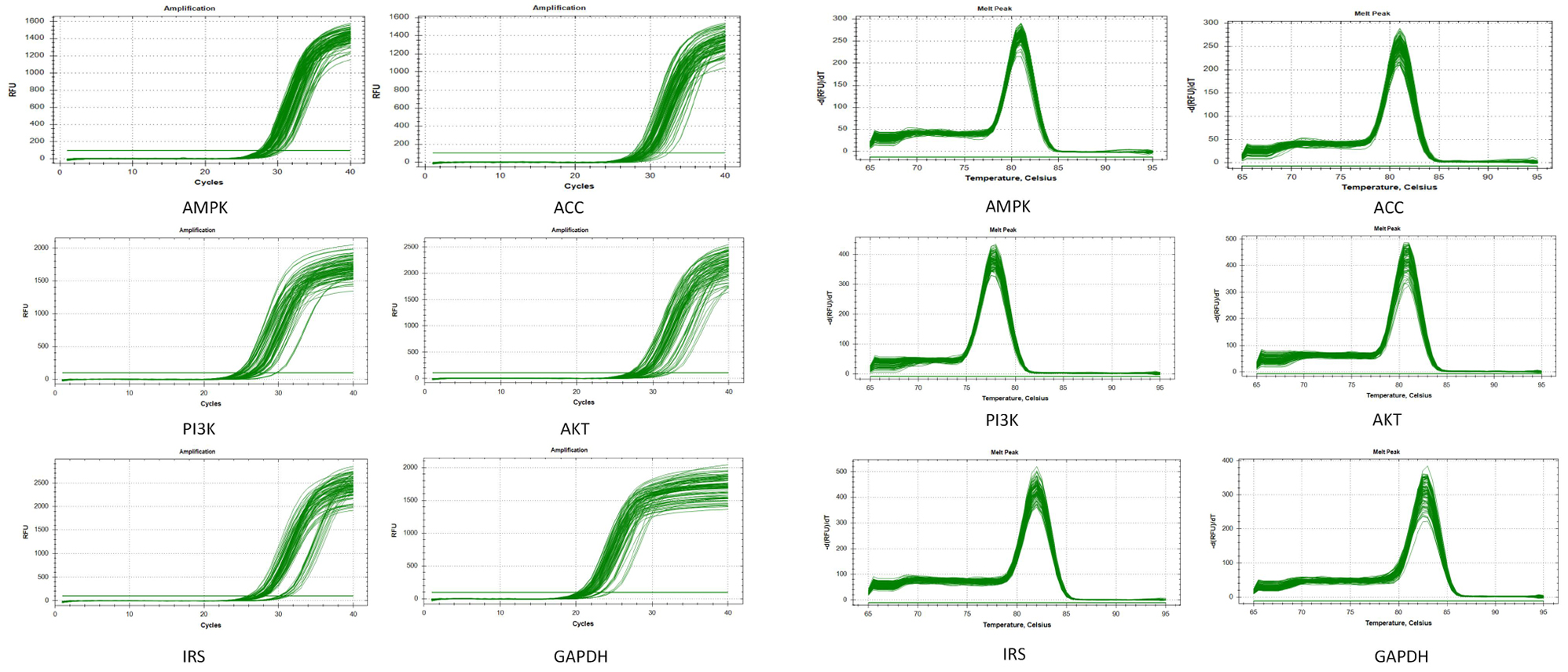
(**a**)Amplification curves of PCR in various groups of mice.(**b**)Melting curves of PCR in various groups of mice

Detection results of the mRNA expression level of ACC. At 1 week of intervention, compared with the Con group, the mRNA expression of ACC in the Mod group increased, with a highly significant difference (*P* < 0.01); compared with the Mod group, the mRNA expression of ACC in the Acu + AMPK antagonist group and the Acu + ACC antagonist group decreased, with a significant difference (*P* < 0.05); at 4 weeks of intervention, compared with the Con group, the Mod group ACC’s mRNA expression increased, with significant difference (*P* < 0.05); compared with the Mod group, the mRNA expression of ACC in the Acu + AMPK antagonist group and Acu + ACC antagonist group decreased, with highly significant difference (*P* < 0.01) (Figs. 6 and 7).

### Comparison of protein expression levels of IRS1, PI3K, AKT, AMPK, and ACC in the liver tissues of mice in each group

**Fig. 8:**
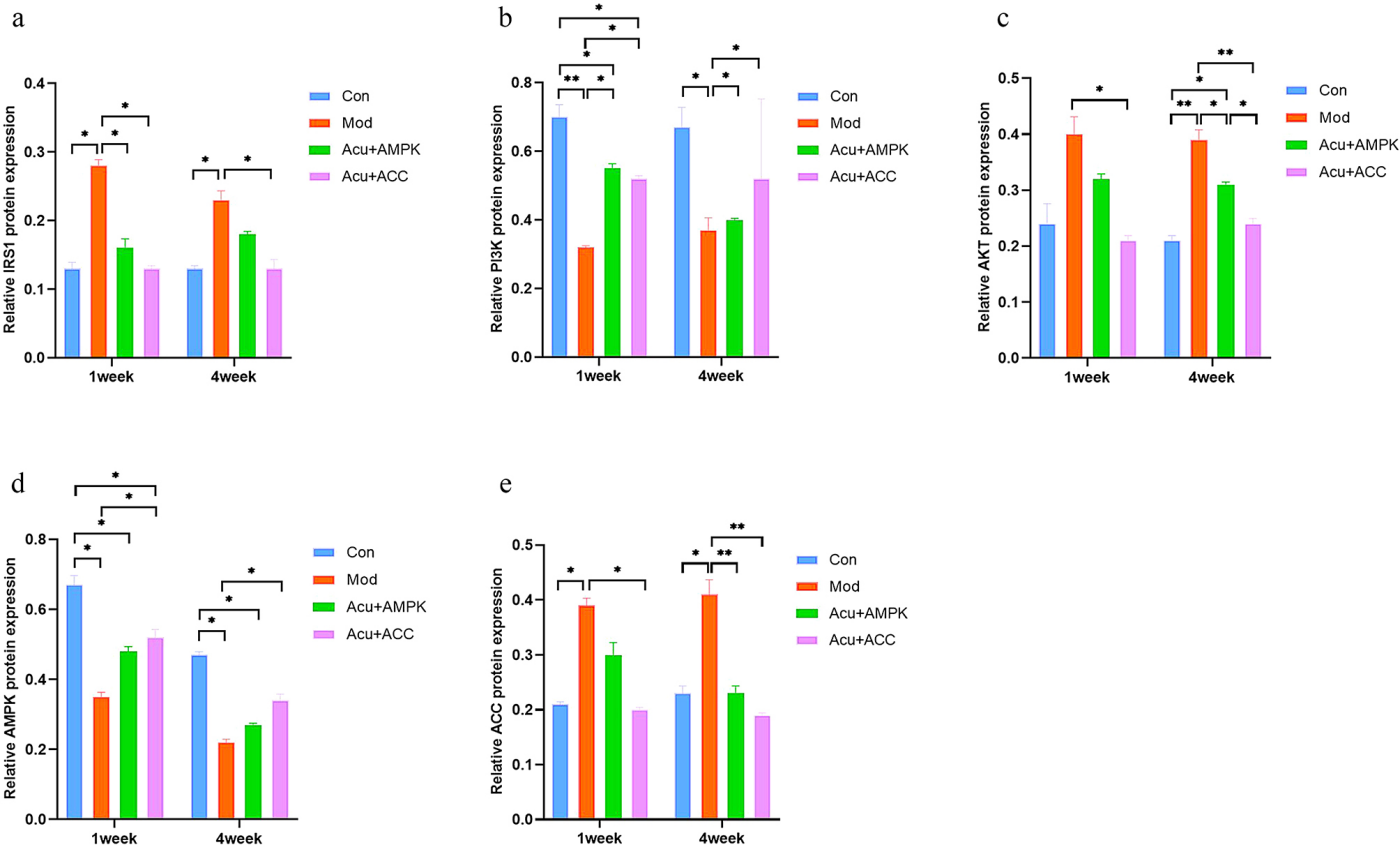
Comparison of protein expression levels of IRS1(**a**), PI3K(**b**), AKT(**c**), AMPK(**d**), and ACC(**e**) in liver tissues of mice in each group. Results are mean value ± SD(*n* = 5).*p < 0.05,**p < 0.01,compared with Con.*p < 0.05,**p < 0.01,compared with Mod.*p < 0.05,**p < 0.01,compared with Acu + ACC

At 1 week of intervention, compared with the Con group, the protein expression of PI3K and AMPK in the Mod group was decreased, the protein expression of IRS1 and ACC was increased, with significant differences (*P* < 0.05), and there was no significant difference in the protein expression of AKT (*P* > 0.05); The protein expression of AMPK in Acu + AMPK antagonist group was decreased with significant difference (*P*<0.05), and there was no significant difference in IRS1, PI3K, AKT, ACC protein expression (*P*>0.05); the protein expression of AMPK in Acu + ACC antagonist group was decreased with significant difference (*P*<0.05), and there was no significant difference in IRS1, PI3K, AKT, and ACC protein expression was not significantly different (*P* > 0.05). Compared with the Mod group, the protein expression of IRS1 in the Acu + AMPK antagonist group was decreased, the protein expression of PI3K was increased, with significant differences (*P* < 0.05), and there were no significant differences in the protein expression of AKT, AMPK, and ACC (*P* > 0.05); and in the Acu + ACC antagonist group, the protein expression of IRS1, AKT, and ACC was decreased, and PI3K, AMPK protein expression increased, with significant difference (*P* < 0.05). At 4 weeks of intervention, compared with the Con group, the protein expression of IRS1, AKT, and ACC in the Mod group was increased, and the protein expression of PI3K and AMPK was decreased, with significant differences (*P* < 0.05); the protein expression of AMPK in the Acu + AMPK antagonist group was decreased, and the protein expression of AKT was increased, with significant differences (*P* < 0.05), and the protein expression of IRS1, PI3K, and ACC protein expression were not significantly different (*P* > 0.05); IRS1, PI3K, AKT, AMPK, and ACC were not significantly different in the Acu + ACC antagonist group (*P* > 0.05). Compared with the Mod group, the protein expression of PI3K in the Acu + AMPK antagonist group was increased, and the protein expression of AKT and ACC was decreased, with significant differences (*P* < 0.05), and there were no significant differences in the protein expression of IRS1 and AMPK (*P* > 0.05); the protein expression of PI3K and AMPK in the Acu + ACC antagonist group was increased, and the protein expression of IRS1 and AKT, ACC protein expression decreased with significant difference (*P* < 0.05). Compared with the Acu + ACC antagonist group, the protein expression of AKT in the Acu + AMPK antagonist group was increased, with a significant difference (*P* < 0.05), and there was no significant difference in the protein expression of IRS1, PI3K, AMPK, and ACC (*P* > 0.05) (Fig. 8).

## Discussion

Among the diseases of glucose and lipid metabolism, T2DM and NAFLD are the most common. NAFLD is a clinical case syndrome characterized by hepatocellular steatosis and lipid accumulation due to fat accumulation in hepatocytes caused by a variety of reasons other than excessive alcohol intake, and it is a kind of metabolic stress liver injury closely related to insulin resistance (IR) and genetic susceptibility. T2DM is a metabolic disease caused by IR and insufficient insulin secretion [30], and patients often exhibit obesity, excessive drinking, polyphagia, polyuria, elevated blood glucose, and lipids in biochemical tests, and severe diabetic complications such as diabetic foot, diabetic fundus changes, and so on. The 2023 edition of the American Diabetes Association’s (ADA) “Standards for the Medical Care of Diabetes Mellitus” guideline emphasizes the need to screen for NAFLD for all patients with T2DM [31]. Studies have confirmed that there is a strong association between T2DM and NAFLD, which often develops synergistically and is prone to the complications of multiple chronic metabolic diseases [32]. Clinical studies have found that both T2DM and NAFLD have metabolic syndrome-related manifestations such as elevated body weight, increased fasting glucose, and dyslipidemia. Studies have shown that IR is the link between the two, contributing to the poor outcome of the disease [33]. Increased IR in the liver of patients with NAFLD reduces insulin activity and insulin sensitivity, which affects glycemic control in T2DM and participates in the disease progression of its major complications, so the difficulty of glycemic control is also elevated in patients with T2DM with comorbid NAFLD [34]. Obesity and IR are two important features of T2DM and also the most important causative factors associated with NAFLD. In this study, we established a mouse model of T2DM with NAFLD and included animals that met the model criteria in the experiment to study the physiological and pathological mechanisms of disorders of glucose-lipid metabolism by observing the body weight, food intake, water intake, changes in blood glucose and blood lipids, hepatic steatosis, as well as the protein and gene indexes related to the AMPK/ACC pathway in mice.

In this study, based on the etiology and pathogenesis of the disease, which is characterized by “spleen deficiency dampness ride, and phlegm and drink blocking the collaterals”, and with the therapeutic principle of “invigorating spleen for eliminating dampness”, we innovatively proposed the application of electroacupuncture with “adjusting internal organs and dredging channelon” to treat abnormalities of glycolipid metabolism in mice with T2DM and NAFLD. T2DM mice with NAFLD have abnormal glucose and lipid metabolism. The group has already confirmed that electroacupuncture treatment of glycolipid metabolism in T2DM with NAFLD mice is reasonable in the experiment through the previous study [35]. Electroacupuncture is a combination of traditional acupuncture and electrical stimulation, which forms electrical circuits between acupoints and the body through different frequencies and waveforms, thereby interfering with the abnormal metabolic state of the body. Studies have shown that electroacupuncture can improve abnormal energy metabolism by reducing oxidative stress, ectopic fat deposition, and changing metabolic flux, thus improving severe metabolic disorders in ZDF rats [2]. Electroacupuncture can also bring pancreatic β-cells into full play, which is known as “pancreatic islet sensitizer” [36], and can better solve the glycolipid metabolism problem of T2DM with NAFLD due to IR. Clinical results show that electroacupuncture can produce stronger stimulation of acupuncture points than traditional acupuncture, which is called the “arrival of qi” in Chinese medicine. In this study, Lung Yu, Spleen Yu, Kidney Yu, Taichong, Tsusanli, Sanyinjiao, and Hegu were selected as the main acupuncture points for treatment. Relying on the therapeutic principle of acupuncture and moxibustion “distal-proximal points combination, inside and outside correspondingly”, it realizes the therapeutic effect of regulating the internal organs, dispersing stagnated liver qi for relieving qi stagnation, regulating the spleen and stomach, resolving turbidity and lowering lipids.

The research group found through the preliminary clinical trials, the use of electroacupuncture treatment of T2DM with NAFLD patients in more than 90 cases, the experimental results show that 89.7% of the patients’ blood glucose and triglyceride levels have been effectively improved. Obesity and excessive food and water intake are typical manifestations of T2DM combined with NAFLD, and the daily activities of the mice in each group were not affected after electroacupuncture stimulation. In this study, we analyzed the physiological indexes of the mice and found that after 4 weeks of electroacupuncture treatment, the body weight, food intake, and water intake of mice in the Acu + AMPK antagonist group and the Acu + ACC antagonist group decreased compared with those in the first week, suggesting that electroacupuncture treatment improved the obesity and excessive food and water intake in T2DM with NAFLD mice. Promote the reduction of clinical symptoms of impaired glucose-fat metabolism.Related studies have shown that subcutaneous fat metabolism affects changes in body weight data as a result of long-term effects, and the limited time available for the present experimental study still needs to be further explored in the future to find out why acupuncture changes body weight by affecting subcutaneous fat metabolic rate [37].

In the spontaneous T2DM with NAFLD model of this study, the mice showed glucose metabolism disorder-related manifestations such as abnormal blood glucose, lipid, and insulin levels. Blood glucose is the main basis for the diagnosis of T2DM, as well as the main indicator for judging the condition and treatment effect. After 4 weeks of electroacupuncture stimulation intervention, the blood glucose level of mice in Acu + AMPK antagonist group and Acu + ACC antagonist group decreased significantly compared with the first week, and the blood glucose values of the two groups were lower than those of the model group, which was attributed to the fact that electroacupuncture could increase the level and ability of glycogen storage in the livers of the db/db mice, and increase the content of hepatic glycogen, which had a certain regulating effect on the glucose metabolism.TG is a diagnostic index of lipid metabolism disturbance, and HDL-C is a major indicator for the diagnosis of T2DM, as well as a main indicator for judging the condition and treatment effect. TG is a diagnostic indicator of lipid metabolism disorder, HDL-C and LDL-C are the forms of lipids’ existence, transportation, and metabolism in the blood, and CHO is an important indicator for judging lipid metabolism, and TG, HDL-C, LDL-C, and CHO are often detected in clinics, which are used for preventing the occurrence of hyperlipidemia and obesity, and at the same time, as a detection indicator after the use of lipid-lowering drugs for treatment. In terms of lipid-related indicators, TG, LDL, and CHO in the Acu + AMPK antagonist group and the Acu + ACC antagonist group decreased continuously from week 1 to week 4, whereas the HDL expression level increased, and this result was attributed to the fact that electroacupuncture could inhibit the abnormalities of lipid metabolism, reduce lipid deposition, and decrease the level of blood lipids. The level of INS is one of the most important indicators for assessing IR. In terms of the increase in INS caused by IR, the situation has significantly improved since the first week, and the INS situation has stabilized in the fourth week but is still lower than the serum INS of the model group, which is attributed to the fact that electroacupuncture can increase insulin sensitivity and alleviate IR. The above results show that electroacupuncture for T2DM and NAFLD mice has a positive significance on glucose and lipid metabolism, which not only improves the existing glucose and lipid metabolism, especially lipid metabolism, but also promotes the increase of beneficial cholesterol, and the hypoglycemic and lipid-regulating effects may be related to the control of the protein pathway to enhance the uptake and utilization of glucose, and to promote the oxidation of fatty acids.

The liver is the main place of fat metabolism, and the improvement of IR level is mainly achieved by controlling blood glucose and regulating the secretion function of adipose tissue. In this study, the HE staining method was applied mainly to observe the histopathological changes of the liver and the PAS method to detect hepatic glycogen synthesis. The microscopic results showed that the degree of hepatic tissue steatosis in the Acu + AMPK antagonist and Acu + ACC antagonist groups was significantly smaller than that in the model group, and the number and range of lipid droplets were significantly reduced. The results of HE staining and PAS staining in this study showed that electroacupuncture was able to reduce the pathological changes of liver tissue, improve steatosis, and increase hepatic glycogen synthesis. This suggests that the electroacupuncture intervention of “adjusting internal organs and dredging channelon” can effectively improve the liver tissue injury of T2DM with NAFLD, which suggests that electroacupuncture may be related to the control of the protein pathway and the inhibition of gluconeogenesis, lipid synthesis, and glycogen synthesis at the same time.

AMP-activated protein kinase (AMPK) is an important energy-sensing enzyme whose activity is regulated by the AMP/ATP ratio and regulates cellular energy homeostasis by inhibiting anabolism and activating catabolic processes. It is the master switch for regulating the energy metabolism of the body and plays a key role in regulating hepatic lipid metabolism, including glucose transport, It plays a key role in the regulation of hepatic lipid metabolism, glucose transport, lipolysis, and gluconeogenesis, and is an important target for the treatment of T2DM, NAFLD, IR, obesity and other diseases. Hepatic AMPK activation can regulate cellular glucose-energy metabolism homeostasis by inhibiting hepatic gluconeogenesis and down-regulating gluconeogenesis genes [38], and AMPK inhibits metabolic pathways such as fat synthesis, glycogenolysis, and protein synthesis, and positively regulates glycolysis and fatty acid oxidation. Studies have shown [39] that AMPK can improve glycolipid metabolism when it is activated and phosphorylated by a variety of upstream kinases. The AMPK family consists of three subunits, AMPKα, AMPKβ, and AMPKγ, with AMPKα as its major subunit [40].AMPK can be activated by phosphorylation of the AMPKα Thr172 site, which regulates its downstream molecules, such as ACC, to reduce triglyceride synthesis and accelerate fatty acid oxidation [41, 42]. Acetyl-CoA carboxylase (ACC) is a rate-limiting enzyme that promotes the synthesis of fatty acids from scratch. It is a crucial factor in the pathogenesis of glycolipid metabolism disorders. Its main function is to catalyze the formation of malonyl CoA from acetyl-CoA carboxylase A, which generates endogenous fatty acids under the action of fatty acid synthases. These fatty acids are eventually stored as triglycerides. The endogenous fatty acids are generated by the latter under the action of fatty acid synthase. These fatty acids are ultimately stored in the form of TG or transported outside the cell in the form of very low-density lipoprotein particles. When the body is in a state of insulin resistance, lipid metabolism is also impaired, and fatty acid synthesis is reduced. This suggests that glucose utilization is impaired and that insulin regulates ACC, which affects fatty acid synthesis [25]. In the previous study, it was found that the activity of ACC is regulated by AMPK, specifically through the phosphorylation of Ser79 and Ser212 on ACC1 and ACC2. This phosphorylation can accelerate lipolysis, inhibit the synthesis of fatty acids and cholesterol, and reduce hepatic lipid accumulation. Additionally, the expression of ACC downstream of AMPK increases when the activity of AMPK is inhibited [43, 44]. The experimental results indicate that the relative protein expression levels of IRS1, AKT, and AMPK were significantly reduced in the Mod group compared to the Con group. Additionally, the relative protein expression levels of PI3K and ACC were significantly increased, which is consistent with the findings of a previous study [45]. These results suggest that the expression of AMPK is affected by the IR situation. Therefore, activation of AMPK/ACC can improve the disorders of glucose-lipid metabolism and jointly promote the disease progression of T2DM with NAFLD.

To explore the regulation of AMPK/ACC signaling pathway-related factors expression by electroacupuncture, this study used Western Blot, qRT-PCR technology, and at the same time, applied target protein antagonist to block the pathway, respectively, to observe the expression levels of IRS1, PI3K, AKT, AMPK, ACC proteins, and their mRNAs in the 1st and 4th weeks of the acupuncture treatment, AKT, AMPK, ACC proteins and their mRNA expression levels were observed respectively after the 1st and 4th weeks of acupuncture treatment. The experimental results showed that compared with the Mod group, the expression levels of IRS1 and AKT proteins in the Acu + AMPK antagonist group and the Acu + ACC antagonist group were significantly reduced, and the relative expression levels of PI3K proteins were significantly increased, which indicated that electroacupuncture could effectively activate the IRS1/PI3K- AKT signaling pathway and effectively reduce the expression level of AKT proteins and their mRNA. AKT signaling pathway effectively improves the signaling pathway activity, and regulates the transport and absorption of glucose in hepatocytes, thereby reducing the peripheral blood glucose concentration and improving the liver IR. Comparison of the results between the Mod group and the Acu + ACC antagonist group showed that the relative expression level of AMPK protein was significantly higher, while comparison of the results between the Mod group and the Acu + AMPK antagonist group showed that the relative expression level of AMPK protein was not significantly different, and the relative expression level of AMPK protein was not significantly different from that of ACC. Comparison results between the Mod group and the Acu + AMPK antagonist group showed that there was no significant difference in AMPK protein expression level, while ACC protein expression level was significantly reduced. The above results indicated that electroacupuncture can down-regulate the expression of ACC protein, AMPK plays a key role in the AMPK/ACC pathway. The results of this experiment are consistent with other studies in this field, such as liraglutide may improve T2DM-associated NAFLD by activating the AMPK/ACC signaling pathway [46]; EA at ST40 and ST36 can significantly improve the liver function of NAFLD rats, and its mechanism is related to the PERK/ATF4/CHOP signal pathway, which can inhibit endoplasmic reticulum stress and protect the liver [47].

In summary, electroacupuncture can improve liver glucose metabolism. Electroacupuncture can improve hepatic glucose metabolism disorders, increase hepatic insulin sensitivity, and improve insulin resistance, indicating that the effect of electroacupuncture on improving glucose and lipid metabolism is clear, but the regulatory mechanism may be complex, and the AMPK/ACC pathway is only one of the possible pathways.

## Conclusions

In this study, db/db mice were used as research objects, and western blot, PCR, and other detection techniques were used to detect pathological changes in liver tissue and the content of AMPK/ACC signaling pathway-related genes and proteins, AMPK, and ACC, to explore the mechanism of the disorders of glucose and lipid metabolism in db/db mice with the electroacupuncture. To explore the mechanism of disorders of glycolipid metabolism in db/db mice, the following conclusion was drawn: “adjusting internal organs and dredging channelon” electroacupuncture can improve the general condition, glycolipid metabolism level, etc., and it has a certain effect on improving and repairing the liver tissues of db/db mice. Electroacupuncture can improve liver IR, affect the expression of AMPK, down-regulate the expression of ACC protein, and play a therapeutic role in T2DM with NAFLD.

## Data Availability

No datasets were generated or analysed during the current study.
